# *QuickStats:* Percentage* of Children and Adolescents Aged 12–17 Years Who Participated in 60 Minutes of Physical Activity Most Days or Every Day,^†^ by Daily Hours of Screen Time Use^§^ — United States, July 2021–December 2023

**DOI:** 10.15585/mmwr.mm7344a5

**Published:** 2024-11-07

**Figure:**
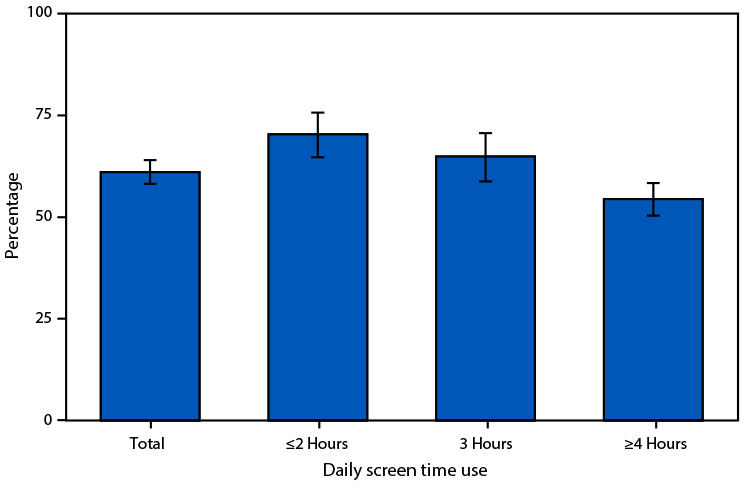
During July 2021–December 2023, 61.1% of children and adolescents reported 60 minutes of physical activity most days or every day. Physical activity decreased with increasing hours of screen time use, from 70.4% among those with ≤2 hours of screen time to 54.4% among those with ≥4 hours of screen time.

**Supplementary Table:** https://stacks.cdc.gov/view/cdc/166706

**Source:** National Center for Health Statistics, National Health Interview Survey-Teen, July 2021–December 2023. https://www.cdc.gov/nchs/nhis/teen.htm

For more information on this topic, CDC recommends the following link: https://www.cdc.gov/physical-activity-basics/guidelines/children.html.

